# Improvement in Clinical Features of L-NAME-Induced Preeclampsia-like Rats through Reduced SERPINA5 Expression

**DOI:** 10.3390/biom13121792

**Published:** 2023-12-14

**Authors:** Shanshui Zeng, Zimeng Liu, Jiaye Yin, Shu Li, Min Jiang, Hongling Yang, Yan Long

**Affiliations:** Department of Laboratory, Guangzhou Women and Children’s Medical Center, Guangzhou Medical University, Guangzhou 510623, China; shanshuiz@stu.gzhmu.edu.cn (S.Z.); 2021210365@stu.gzhmu.edu.cn (Z.L.); 13002066233@163.com (J.Y.); shuli2015@whu.edu.cn (S.L.); jiangmin2011@126.com (M.J.); hlyang62@126.com (H.Y.)

**Keywords:** pre-eclampsia, SERPINA5, placenta, rat, L-NAME

## Abstract

Pre-eclampsia (PE) is a severe pregnancy disorder that poses a significant health risk to both mother and fetus, with no preventive or therapeutic measures. Our previous research suggested an association between elevated SERPINA5 levels and PE features. This study investigated whether SERPINA5 could be a potential therapeutic target for PE. We established PE-like features in pregnant rats using L-NAME (75 mg/kg/d) treatment. Adenoviruses carrying overexpressed or suppressed SERPINA5 genes were intravenously injected into these PE rats on the fifth and seventh days of pregnancy. We evaluated the rats’ systolic blood pressure, urine protein concentration, and placental and fetal metrics and histology. Placental gene expression following SERPINA5 overexpression was evaluated using mRNA sequencing. The L-NAME-induced PE rat model observed a significant increase in placental and peripheral SERPINA5 levels. The overexpression of SERPINA5 exacerbated L-NAME-induced hypertension and proteinuria in pregnant rats. A histology examination revealed a smaller placental junctional zone in L-NAME + overexpressing rats. Placental gene expression analysis in the L-NAME + overexpressing group indicated increased coagulation activation. L-NAME-induced hypertension and proteinuria were mitigated when SERPINA5 expression was suppressed. Additionally, placental development was improved in the SERPINA5-suppressed group. Our findings suggested that SERPINA5 may worsen L-NAME-induced PE-like features by promoting the activation of the coagulation cascade. Therefore, reducing SERPINA5 expression could potentially serve as a therapeutic strategy for PE.

## 1. Introduction

Pre-eclampsia (PE) is a condition that occurs after the 20th week of gestation, characterized by newly developed hypertension (diastolic blood pressure ≥ 90 mm Hg and/or systolic blood pressure ≥ 140 mm Hg) and proteinuria (≥0.3 g/day) or the dysfunction of multiple organs [1,2]. This condition affects 3–5% of pregnancies worldwide, leading to significant maternal and fetal morbidity and mortality [3]. Maternal complications include eclampsia, liver, kidney, and coagulation abnormalities, placental abruption, and cardiovascular incidents [4,5,6], while fetal complications may involve growth restriction, fetal distress, preterm delivery, and stillbirth [7,8]. Additionally, PE increases the risk of cardiovascular and end-stage renal disease post-pregnancy [9]. The only definitive treatment for PE is the delivery of the fetus and placenta. The diverse pathophysiological causes of PE and the incomplete understanding of its etiology hinder the development and implementation of prognostic and therapeutic strategies. Consequently, an in-depth exploration of the psychophysiology pathways associated with PE is crucial for improving prevention and detection methods.

The pathogenesis of PE is notably heterogeneous, with theories attributing the disease to excessive inflammation, oxidative stress, metabolic disorders, and apoptosis [2,10,11]. The current understanding posits that PE develops in preclinical and clinical stages [12]. Key pathological changes, such as inadequate trophoblast invasion and spiral artery remodeling, have been identified as crucial factors in the preclinical phase [12,13]. These changes result in placental ischemia and hypoxia, triggering the release of anti-angiogenic, inflammatory, and immune factors. This cascade of events subsequently leads to widespread vascular endothelial dysfunction in the clinical phase of PE. However, our understanding of PE’s preclinical phase remains incomplete due to the disease’s heterogeneity and limitations in research methodologies. Recent studies have underscored the variability in expression patterns of disease-related factors throughout pregnancy [14].

Our previous research suggested that serum levels of SERPINA5 were elevated five-fold before the onset of PE, potentially promoting PE progression by inhibiting uPA/uPAR signaling and thus suppressing EVT invasion [15,16]. SERPINA5, a protein C inhibitor (PCI), is a serine protease inhibitor. It plays a critical role in limiting the activity of the anticoagulant protein C. SERPINA5 is involved in many biological processes, including inflammation, coagulation, and responses to elevated platelet cytoplasmic Ca^2+^. An in vivo study demonstrated that SERPINA5 overexpression could induce PE-like features in multiple organs in rats [16]. However, the organ or tissue which secretes SERPINA5 protein during PE pregnancy remains uncertain, and whether it could serve as a potential intervention target. Nω-nitro-L-arginine methyl ester hydrochloride (L-NAME), an arginine analog that inhibits nitric oxide (NO) production, can obstruct acetylcholine-induced diastole and elevate arterial blood pressure [17,18]. Administering L-NAME during pregnancy could induce PE features such as hypertension, proteinuria, and fetal dysplasia in rats [19,20]. This model also facilitates the observation of placental dysplasia in placental pathology, resulting from reduced trophoblast invasion [20]. Our current hypothesis was that rats overexpressing SERPINA5 exhibit reduced trophoblast invasion and remodeling of spiral arteries. Therefore, we utilized the L-NAME-induced PE model to evaluate SERPINA5 as a potential therapeutic target.

In this study, we administered adenovirus (ADV) containing the SERPINA5 gene to rats, creating a pregnant rat model with elevated SERPINA5 expression. We then examined maternal serum SERPINA5 levels to determine their correlation with clinical features of PE and to investigate potential pathways. Additionally, we reduced SERPINA5 expression to assess whether it could alleviate the features of L-NAME-induced PE in pregnant rats.

## 2. Materials and Methods

### 2.1. Animal

The animal research protocol was approved by the Animal Care and Use Committee of the Guangdong Experimental Animal Center, China (Approval ID: B201906-10). We acquired forty female and twenty male Sprague-Dawley rats, aged six to eight weeks, from the Guangdong Experimental Animal Center. These rats were accommodated in a pathogen-free environment. The female rats were given a two-week adjustment period until they reached ten weeks of age. The housing conditions were kept at 22 ± 2 °C with 55 ± 15% humidity under a 12-h light-dark cycle. The rats were given unlimited access to food and water.

### 2.2. Animal Experimental Procedures

Animal experiments were conducted with female and male rats (2:1) housed together. Pregnancy was confirmed by finding sperm in the vaginal smear. Pregnant rats were randomly allocated to one of five treatment groups: negative control (NC), L-NAME treated, L-NAME + ADV-SERPINA5-overexpression (OE), L-NAME + OE-control, L-NAME + ADV-SERPINA5-knockdown (KD), and L-NAME + KD-control. Tail blood pressure was measured, and serum and urine were collected every four days. On day 20 of pregnancy, the rats were euthanized, and the fetus number and weight and liver, kidney, and placental tissues were recorded.

The groups were treated as follows:

(1) The NC group (*n* = 6) was given 200 μL/day saline solution from day 9 to day 19 of gestation.
(2) The L-NAME treated group (*n* = 8) was assigned L-NAME(200 μL, 75 mg/kg/day via the subcutaneous, from day 9 to day 19 of gestation) [21,22].
(3) L-NAME + OE group (*n* = 7): Recombinant ADV with the SERPINA5 gene (ADV-pCMV-SERPINA5) was used to overexpressed SERPINA5 in vivo. ADV-pCMV-SERPINA5 (200 μL, 1 × 10^9^ PFU/rat) was administered to rats in GD5 and GD7 by tail injection [23]. At the same time, this group of rats received L-NAME treatment at a dose of 75 mg/kg/day, injected subcutaneously from day 9 to day 19 of gestation. One of the rats died after two times of ADV injections, so only 7 rats were included in the results. ADV was made by GeneChem Ltd. (Shanghai, China).
(4) L-NAME + OE-control (*n* = 6): ADV-pCMV-empty was used as a control for SERPINA5 in vivo expression. This group of rats also received L-NAME treatment, as described above.
(5) L-NAME + KD (*n* = 6): In vivo gene knockdown was performed using an ADV vector. A shRNA fragment targeting the rat SERPINA5 gene (NM_001244739) was cloned into the PLko-U6-MCS-CAG-EGFP vector. Knockdown vectors (200 μL, 1 × 10^9^ PFU/rat) were given via tail injection on GD5 and GD7. As described above, these rats also received L-NAME at 75 mg/kg/day subcutaneously.
(6) L-NAME + KD-control (*n* = 6): An empty PLko-U6-MCS-CAG-EGFP vector was used as a control for in vivo gene knockdown. The control vector (200 μL, 1 × 10^9^ PFU/rat) was given via tail injection on GD5 and GD7. As described above, these rats also received L-NAME at 75 mg/kg/day subcutaneously.

### 2.3. Blood Pressure Measurement

Blood pressure was measured on gestation days 0, 8, 12, 16, and 20 using a non-invasive blood pressure monitoring system (BP-2010A, Softron, Beijing, China). The rats were warmed using a heating jacket (40 °C) for five minutes to improve tail blood circulation. The sensor was placed at the base of the tail, and measurements were initiated once the blood pressure curve stabilized. This process was repeated five times, and the average value was calculated.

### 2.4. Sample Collection

Blood and urine were collected on gestation days 0, 8, 12, 16, and 20. Approximately 0.5 mL of blood was collected from the tail tip (serum was obtained via centrifugation at 3000× *g*), and bleeding was halted through applied pressure. Each rat was individually housed in a metabolic cage (TSE system, Chesterfield, MO, USA), and urine was collected over 12 h (8 p.m. to 8 a.m.), during which the rats had regular access to food. The collected urine was centrifuged, and the supernatant was stored for subsequent analysis. Post-euthanasia, the liver, kidney, placenta, and fetal rats were extracted. The placentas and fetal rats were weighed. Tissues were thrice rinsed in PBS buffer to eliminate erythrocytes, then fixed in 4% paraformaldehyde for 24 h before paraffin embedding.

### 2.5. Semi-Quantification of Serum SERPINA5 Levels

Rat serum SERPINA5 levels were quantified using an enzyme-linked immunosorbent assay (ELISA). The SERPINA5 antibody (10673-1-AP, Proteintech Group, Wuhan, China), diluted in carbonate buffer, was initially added to the enzyme-labeling plate and incubated overnight at 4 °C. The plates were then washed with TBST buffer for 5 min, repeated thrice. Subsequently, 100 μL of the test serum was added to the wells and incubated for two hours, followed by a 5-min wash repeated three times. The diluted HRP-conjugated detection antibody (1:1000) was added and incubated for one hour. After washing, the TMB substrate was added and set for 30 min before the reaction was terminated with a termination solution. Absorbance was measured at 450 nm using a plate reader. A diluted antigen (1266-PI, R&D systems, Minneapolis, MN, USA) was a positive control, and PBS buffer was used as a negative control.

### 2.6. Urine Protein Measurement

Rat urine protein was measured using a urine microalbumin assay kit (Beijing Strong Biotechnologies, Beijing, China) on an automatic biochemical analyzer (i7600, Hitachi, Tokyo, Japan). Proteinuria was calculated by multiplying the protein concentration by the 12-h urine output.

### 2.7. RNA Sequencing

Majorbio Biotech Co. (Shanghai, China) facilitated RNA isolation, cDNA library construction, and sequencing. Total placental RNA was extracted using Trizol (Thermo Fisher Scientific, Waltham, MA, USA), and the concentration and purity of the RNA were assessed using Nanodrop2000 (Thermo Fisher Scientific, Waltham, MA, USA). The RIN value was determined by Agilent 2100 (Agilent Technologies, Inc., Santa Clara, CA, USA). The Illumina TruseqTM RNA sample prep Kit was employed for library construction. Transcriptome sequencing was executed via paired-end sequencing on the Illumina Novaseq 6000 sequencing platform (Illumina, San Diego, CA, USA). Raw data (raw reads) were filtered to yield clean reads, which were then analyzed by aligning them to the reference genome using Top Hat2 software (v2.0.12). Differentially expressed genes (DEGs) were identified using the DESeq2 package, with a threshold of |log2FC| ≥ 1 & *p*-value < 0.05. These DEGs were then subjected to GO and KEGG pathway enrichment analysis using the ClusterProfiler (v4.0) package. Heat maps were generated with the pheatmap (v1.0.8) package. Gene set enrichment analysis was conducted using GSEA software (v4.3.2) (https://www.broadinstitute.org/gsea/, accessed on 15 June 2023) [24].

### 2.8. Histology

Immunohistochemistry (IHC) was performed as before [25], using SERPINA5 antibodies (10673-1-AP, Proteintech Group, Wuhan, China). Five images were taken for each section, and staining was quantified using Image J software (v 1.53t). To evaluate the histopathology of the placenta and kidney, H&E staining of the placental sections and H&E, PAS, and Masson staining of the kidney sections were performed. H&E, PAS, and Masson staining were performed as described earlier [26]. Five randomly selected fields of view were photographed for a histological assessment for each section. Placenta and renal histology were assessed blindly by an experienced pathologist. Histological changes in the placenta were quantified by the ratio of the labyrinthine to the junction zone. Mesangial proliferation was evaluated by H&E and PAS staining and scored according to the degree from 0 to 4 (0, absent; 1, mild; 2, mild-moderate; 3, moderate; 4, severe). Endocapillary hypercellularity is assessed by H&E staining on a scale of 0–4 (0 = absent, 1 ≤ 25 percent present, 2 = 25–50 percent present, 3 = 50–75 percent present, 4 ≥ 75 percent of sections present). Interstitial fibrosis was assessed by Masson staining with a score of 0–3 (0 ≤ 5%, 1 = 5–10%, 2 = 11–25%, 3 ≥ 25%).

### 2.9. Statistical Analysis

A *t*-test was used to compare the continuous variables between the two groups. One-way ANOVA was used to compare the data between multiple groups. GraphPad Prism (version 6.0) software was used for statistical analysis. The results were expressed as mean ± standard error of the mean (SEM). *p* < 0.05 was considered statistically significant.

## 3. Results

### 3.1. Serum SERPINA5 Increased in the PE Model Induced by L-NAME

**Figure 1:**
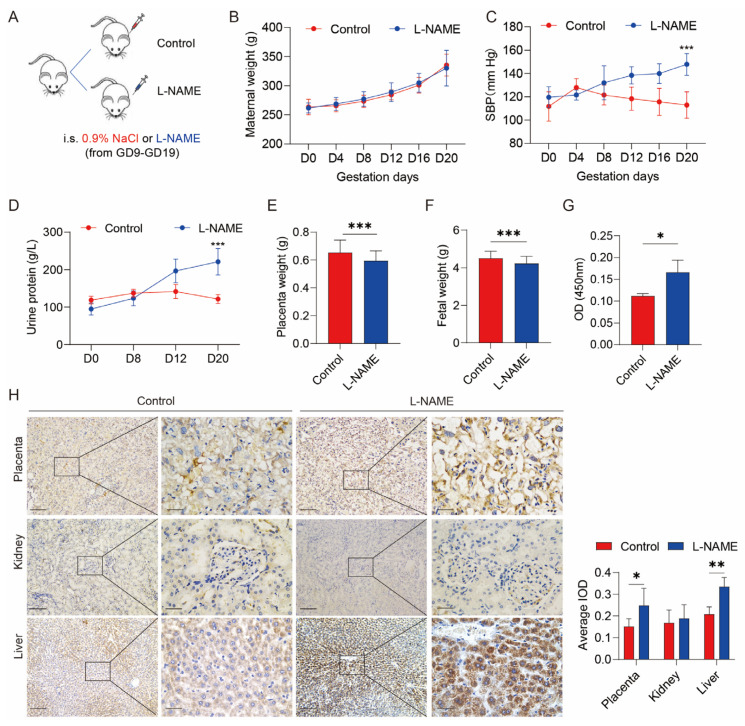
SERPINA5 expression is increased in the L-NAME-induced PE model. (**A**), Pattern diagram of experimental animal procedures, including the L-NAME group (*n* = 8) and control group (*n* = 6). (**B**), Weight increase in female rats during pregnancy. (**C**), Changes in systolic blood pressure in rats during pregnancy. (**D**), Changes in urinary protein concentration in rats during pregnancy in the L-NAME and control groups. (**E**), Placenta weight of L-NAME and control group. (**F**), Fetal weight of L-NAME and control group. (**G**), L-NAME treatment increased serum SERPINA5 levels in pregnant rats. (**H**), SERPINA5 was staining in rat liver, kidney, and placenta. L-NAME increased SERPINA5 expression in the placenta and liver. Scale bar: 200 μm (**left**), 50 μm (**right**). i.s. = injected subcutaneously. * *p* < 0.05, ** *p* < 0.01, *** *p* < 0.001.

A rat PE model (L-NAME group) was established through the tail vein injection of L-NAME (75 mg/kg/day) from gestation day 9 to day 19, with an equivalent volume of 0.9% NaCl injection serving as a negative control (NC group). The model was established successfully upon the observation of hypertension (systolic blood pressure ≥ 140 mmHg) and proteinuria (urinary protein concentration >160 μg/mL). Figure 1A represents a schematic of the experimental animal procedure. Both the L-NAME and control groups exhibited a gradual increase in body weight during pregnancy, with no significant difference (Figure 1B). On gestation days 20, systolic blood pressure was significantly higher in the L-NAME group compared to the control group (Figure 1C, *p* < 0.05). On gestation day 20, urinary protein levels were significantly higher in the L-NAME group compared to the control group (Figure 1D). The L-NAME group demonstrated lower placental and fetal birth weights (Figure 1E,F). Therefore, the PE rat model was successfully established.

**Figure 2:**
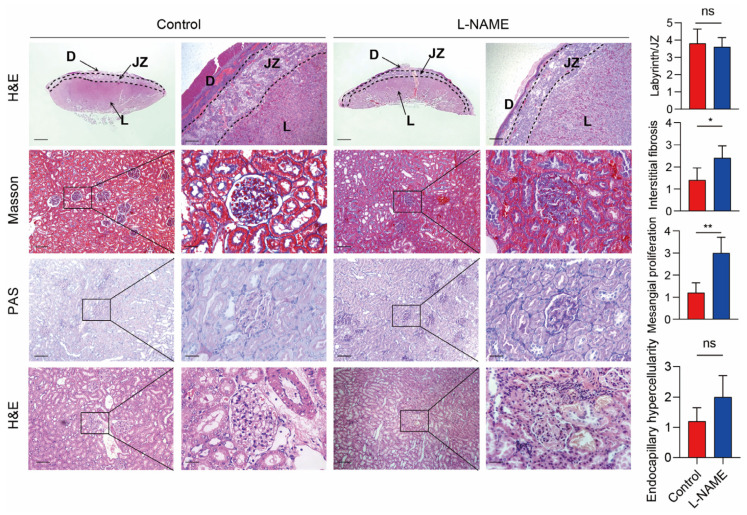
Histopathology analysis of placenta or kidney of L-NAME and control rats. Scale bars: 200 μm (**left**) and 50 μm (**right**). The right panels were the statistical values from six rats per group. The area ratio of the placental labyrinth to the junction area was calculated in placental histology. For renal histology, mesangial proliferation, endocapillary hypercellularity, and interstitial fibrosis were assessed. D = decidua; JZ = junction zone. * *p* < 0.05, ** *p* < 0.01.

An ELISA assay evaluated serum SERPINA5 levels in L-NAME and control rats at gestation day 20. Compared to the control group, serum SERPINA5 levels were significantly higher after L-NAME treatment (Figure 1G), suggesting a correlation between SERPINA5 and PE features. Previous studies have identified human SERPINA5 in the placenta, kidney, and liver; therefore, we examined SERPINA5 expression in these organs following L-NAME administration in rats. In the control group, SERPINA5 was expressed in the placenta and liver, with minimal detection in the kidney (Figure 1H). After L-NAME treatment, SERPINA5 expression significantly increased in the placenta labyrinth, liver, and kidney tissues (Figure 1H). These results support our hypothesis that the placenta partially secretes elevated SERPINA5 in PE pregnancies. Consistent with previous studies, L-NAME treatment resulted in placental dysplasia, characterized by an increased ratio of the placenta labyrinth to the junctional zone (Figure 2). In addition, we observed mesangial proliferation and increased glomerular fiber deposition after L-NAME treatment compared to the control group.

### 3.2. SERPINA5 Overexpression Aggravates L-NAME-Induced PE-like Conditions

**Figure 3:**
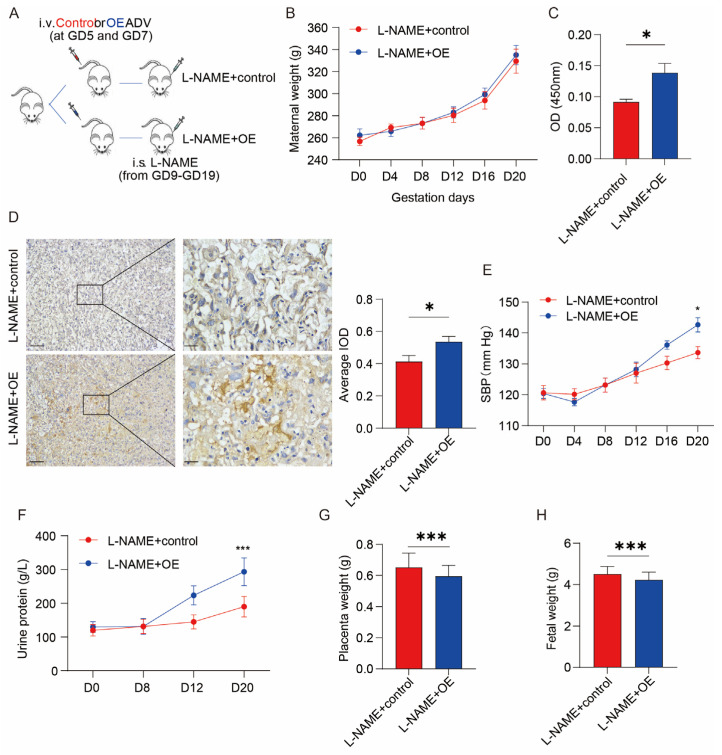
SERPINA5 overexpression promotes L-NAME-induced PE-like features. (**A**), Diagram showing experimental animal methods, comprising L-NAME + OE (*n* = 8) and L-NAME + control (*n* = 6) groups. (**B**), Body weight of female rats during pregnancy. (**C**), Serum SERPINA5 levels in L-NAME + control and L-NAME + OE group. (**D**), SERPINA5 overexpression increased the level of SERPINA5 in the rat placenta compared with L-NAME + control. (**E**), Changes in systolic blood pressure during pregnancy in rats. (**F**), Changes in urinary protein concentration during pregnancy in female rats in the L-NAME + OE group and the L-NAME + control group. (**G**), Placental weights of the L-NAME + OE and L-NAME + control groups. (**H**), Fetal weights of L-NAME + OE and L-NAME + control groups. i.s. = injected subcutaneously. i.v. = injected intravenously. OE = overexpression. * *p* < 0.05, *** *p* < 0.001.

**Figure 4:**
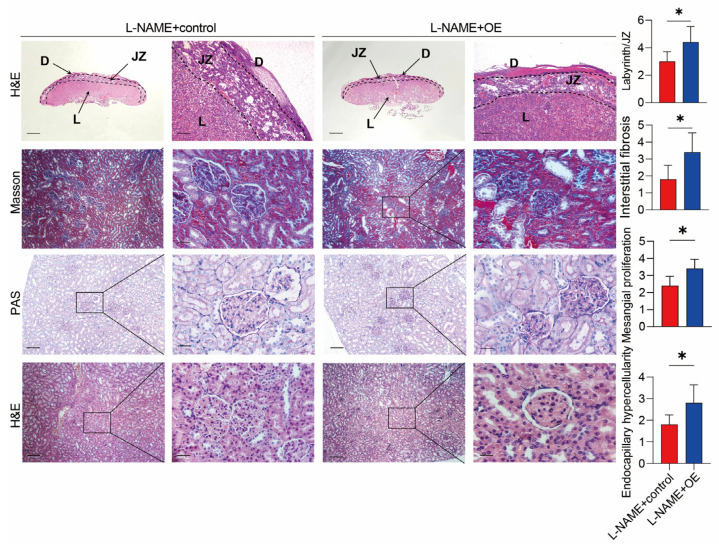
Histopathology analysis of the placenta and kidney of L-NAME + OE and L-NAME + control rats (*n* = 6/group). Scale bars: 200 μm (**left**) and 50 μm (**right**). The right panel was the statistical values from six rats per group. The area ratio of the placental labyrinth to the junction area was calculated in placental histology. For renal histology, mesangial proliferation, endocapillary hypercellularity, and interstitial fibrosis were assessed. D = decidua; JZ = junction zone. * *p* < 0.05.

We used L-NAME to induce PE features in rats and overexpressed SERPINA5 at the same time. Figure 3A represents the course of the animal experiments. The L-NAME + overexpression group (OE) and the control group showed a gradual increase in body weights (Figure 3B). The L-NAME + OE group showed significantly higher levels of serum SERPINA5, systolic blood pressure (SBP), and urinary protein (Figure 3C–F). The number of offspring in the L-NAME + OE group was also lower (Figure 3G,H). The L-NAME + OE group also had fewer offspring and lower fetal and placental weights (Figure 3G,H). The L-NAME + OE group had fewer junctional zones, an increase in the ratio of placental labyrinths/functional zones, a denser tissue structure, and a decrease in vacuoles in the placenta. Renal staining showed the tunica, thickening of the basement membrane, and deposition of interstitial fibrous material (Figure 4). These findings suggest that SERPINA5 overexpression impairs placental and renal function, exacerbating PE in rats.

### 3.3. Changes in the Coagulation Pathway Mainly Led to PE in SERPINA5-Overexpressing Rats

**Figure 5:**
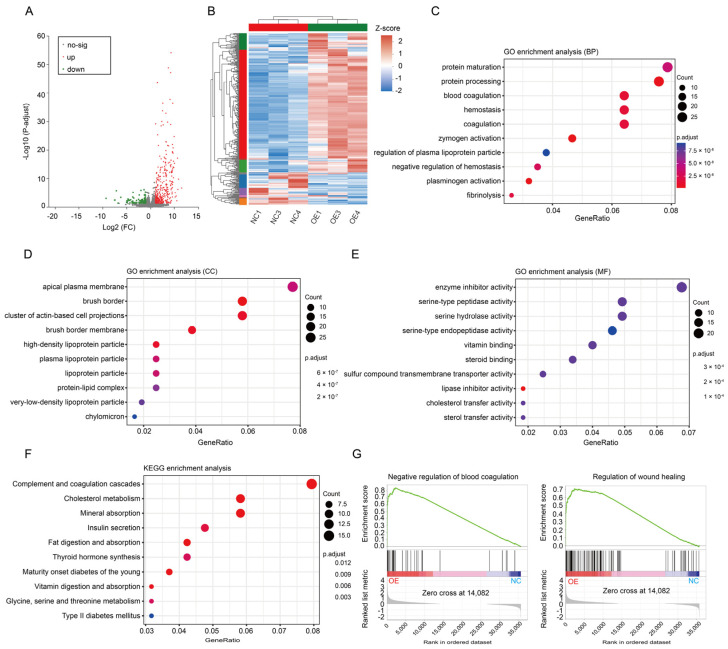
Analysis of placental gene expression in SERPINA5-overexpressing rats (*n* = 3/group). (**A**), Analysis of differential expression gene in the OE and control placentas by volcano plot. (**B**), Placental gene expression profile. Differentially expressed gene clustering made distinguishing the OE group from the control group possible. (**C**–**F**), Gene ontological term clustering analysis of differential expression gene. (**G**), Gene Set Enrichment Analysis (GSEA) of differential expression gene.

To further investigate the impact of SERPINA5 on the placentas of pregnant rats, we employed transcriptome sequencing and bioinformatics analysis on the placentas from SERPINA5-overexpressing and control rats. Principal Component Analysis (PCA) demonstrated that the sequencing results from the overexpression and control groups could be distinctly classified into two groups, with reliable intra-group concordance. With a fold change (FC) >2 and an adjusted *p*-value < 0.05, we identified 270 differentially expressed genes (DEGs) in the placenta of the overexpression group compared to the control group, with 247 up-regulated and 23 down-regulated (Figure 5A). Heat map clustering analysis revealed significant differences between the two placental transcriptomes, with these differences better distinguishing the overexpression group from the control group (Figure 5B). The overexpression of SERPINA5 was associated with coagulation, fiber deposition, and serine protease activity in the placenta according to Gene Ontology (GO) enrichment analysis (Figure 5C–E). Kyoto Encyclopedia of Genomes (KEGG) enrichment analysis (TOP 10) showed that SERPINA5 affects complement and coagulation cascades (Figure 5D). To ascertain whether the GO pathway was differentially enriched between the overexpression and control groups, we conducted an analysis using the Gene Set Enrichment Analysis (GSEA) approach based on the Normalized Enrichment Score (NES) and *p*-values. As depicted in Figure 5G, the overexpression group significantly enriched the GO terms related to coagulation and wound healing regulation. These findings suggest that SERPINA5 overexpression may influence the progression of PE by affecting coagulation, fibrosis, and wound healing pathways.

### 3.4. Inhibiting SERPINA5 Expression Alleviates PE Features

**Figure 6:**
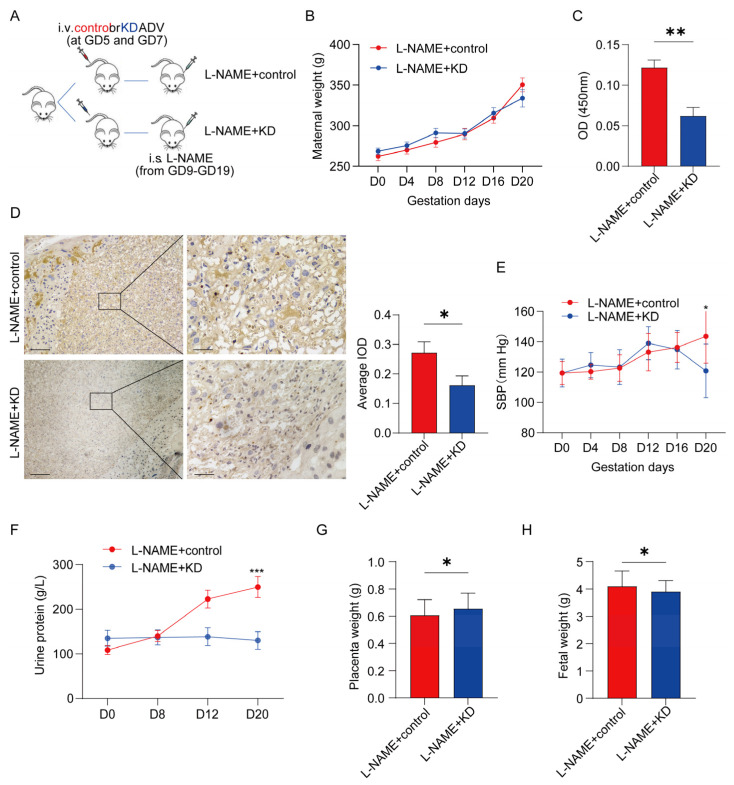
Inhibition of SERPINA5 expression ameliorated L-NAME-induced PE-like features. (**A**), Diagram of experimental animal procedures, including L-NAME + KD group (*n* = 6) and L-NAME + control group (*n* = 6). (**B**), Weight increase condition of female rats during pregnancy. (**C**), Adenovirus carrying the inhibited gene attenuated SERPINA5 levels in rat serum compared to L-NAME+ controls. (**D**), Adenovirus carrying the inhibited gene attenuated SERPINA5 levels in rat placenta compared to L-NAME+ controls. Scale bars: 200 μm (**left**) and 50 μm (**right**). (**E**), Changes in systolic blood pressure in female rats during pregnancy. (**F**), Changes in urinary protein concentration in female rats during pregnancy in the L-NAME + KD and L-NAME + control group. (**G**), Placenta weight of L-NAME + KD and L-NAME + control group. (**H**), Fetal weight of the two groups. i.s. = injected subcutaneously. i.v. = injected intravenously. OE = overexpression. * *p* < 0.05, ** *p* < 0.01, *** *p* < 0.001.

**Figure 7:**
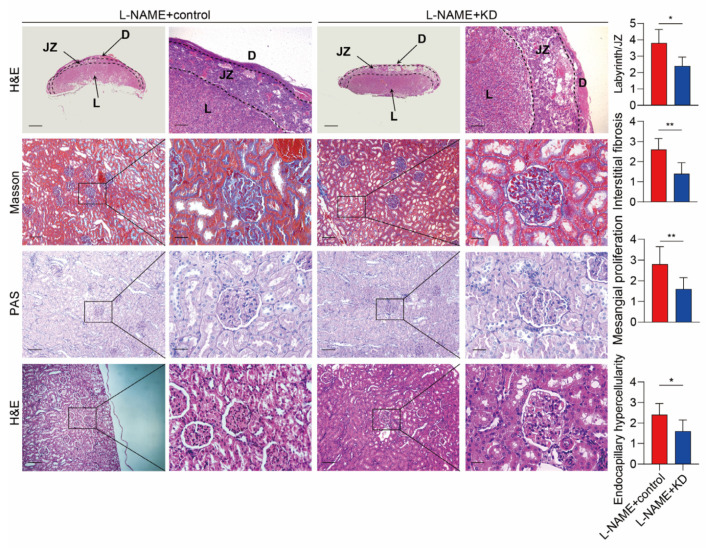
Histopathology analysis of the placenta and kidney of L-NAME + KD and L-NAME + control rats (*n* = 6/group). Scale bars: 200 μm (**left**) and 50 μm (**right**). The right panel was the statistical values from six rats per group. The area ratio of the placental labyrinth to the junction area was calculated in placental histology. For renal histology, mesangial proliferation, endocapillary hypercellularity, and interstitial fibrosis were assessed. D = decidua; JZ = junction zone. * *p* < 0.05, ** *p* < 0.01.

We hypothesized SERPINA5 as a potential PE therapeutic target. Using an L-NAME-induced PE model, we inhibited SERPINA5 expression with an adenovirus carrying short hairpin mRNA. Figure 6A presents a schematic of the animal procedure. No significant differences in weight gain were observed between the L-NAME + KD and control groups (Figure 6B). SERPINA5 levels decreased more than two-fold in the serum and placental tissues of pregnant rats in the L-NAME + KD group (Figure 6C,D). This group also showed reduced SBP from days 12 to 20 and lower urine protein levels (Figure 6E,F). The L-NAME + KD group had lower placental weight but higher fetal birth weight, suggesting that SERPINA5 reduction may alleviate PE-like features (Figure 6G,H). A histological assessment showed improved L-NAME-induced placental and renal changes after the inhibition of SERPINA5 (Figure 7).

## 4. Discussion

Previous studies have demonstrated a correlation between increased SERPINA5 levels and decreased trophoblast cell invasiveness. Additionally, overexpressing SERPINA5 during gestation may cause clinical features in rats that resemble PE [15,16]. The overexpression of placenta-derived SERPINA5 was further demonstrated in this study to cause the dysregulation of blood coagulation, which was linked to anomalous placental development and pathological alterations in the kidneys. On the other hand, L-NAME-induced hypertension and proteinuria were somewhat alleviated, and fetal growth was recovered in rats by preemptively suppressing SERPINA5 expression. According to these results, SERPINA5 could be a therapeutic target for PE intervention and may have a role in the pathophysiology of PE.

SERPINA5 levels have increased dramatically during the preclinical phase of PE and may be related to placental development in the early stages of pregnancy [15]. We hypothesized that the placenta partly caused the serum SERPINA5 to rise during PE. The expression regulation of SERPIN family members (e.g., SERPINA3) is partly based on epigenetic mechanisms and has been associated with PE disease [27]. SERPINA5 relates to male reproductive function and tumor suppression and has been found in the human reproductive tract, liver, and kidney [28,29]. In contrast to humans, SERPINA5 appears to be primarily expressed in the reproductive system of rats. In this work, we established a rat PE model using L-NAME injections, and we discovered that SERPINA5 expression was enhanced in the placenta. We similarly noticed elevated SERPINA5 expression in the liver and kidneys of PE rats but to different degrees. Our earlier research has shown that during severe inflammation, HTR-8/SVneo cells can express SERPINA5 [16]. The increased expression of SERPINA5 in response to the pathophysiological changes in PE is further supported by our study. Curiously, studies have shown that SERPINA5 expression in PE-delivered placentas appears to be no different or even reduced from healthy individuals [30]. We speculate that the possible reason for this is that the expression of SERPINA5 changes with gestation; therefore, longitudinal cohort studies are needed to assess changes in SERPINA5.

The overexpression of SERPINA5 exacerbates L-NAME-induced proteinuria, gestational hypertension, and unfavorable pregnancy outcomes such as fetal growth restriction and miscarriage. After SERPINA5 overexpression, we saw a drop in placental weight in rats, possibly caused by placental dysplasia. PE is a human-only illness that does not impact other species. Therefore, studying the equivalent placental anomalies described in people using animal models that demonstrate substantial trophoblastic invasion during normal pregnancy is essential. The deep intravascular and interstitial layers are permeable to rat placental trophoblast infiltration. Interstitial invasion mimics the consequences of human intravascular trophoblast-associated spiral arterial remodeling, albeit before intravascular invasion [11]. The decidua-invading glycogen-positive cells and trophoblasts, including large trophoblasts, make up the junctional zone of the rat placenta [11,31]. It is thought that trophoblasts’ capacity for invasion is reflected in the size of the placental junctional zone [11,32]. SERPINA5 may impact trophoblast function, as evidenced by the unique placental morphological characteristics (a decreased labyrinth region and an increased junctional zone) observed in L-NAME + OE rats.

We previously found that high levels of SERPINA5 induced PE-like features in rats [16], which could explain why SERPINA5 overexpression exacerbated PE features in L-NAME-induced model rats. Moreover, we detected pathological changes in rat kidneys following SERPINA5 overexpression, akin to previously established PE models [33,34]. The distribution of SERPINA5 in the kidney remains largely unknown. Research indicates that in humans, SERPINA5 is primarily produced in the proximal tubular epithelium of the kidney [35], while it is rare in the rat kidney [36]. It has been proposed that SERPINA5 may inhibit uPA activity in the urinary tract, as human urine contains a significant amount of SERPINA5 bound to uPA [35]. Our results do not confirm whether the renal alterations in rats are a direct effect of SERPINA5 or secondary to pre-eclampsia-like features. Therefore, future studies need to elucidate the role of SERPINA5 in the kidney.

The sequencing analysis of the placental transcriptomes in the SERPINA5 overexpression and control groups revealed that differentially expressed genes were associated with alterations in the coagulation pathway. The coagulation system activation links SERPINA5 with PE features [37]. Healthy pregnancies typically exhibit hypercoagulability, characterized by diminished endogenous anticoagulation and fibrinolytic pathways and heightened procoagulant activity. This hypercoagulable state may prevent excessive bleeding during delivery [37]. In contrast, the coagulation cascade is highly activated in PE pregnancies [38]. The maternal endothelium may be directly or indirectly affected by the placental chemicals released into the maternal bloodstream or placental cell debris entering the circulation. These structural and functional changes alter the vascular response to vasodilator medications, activate the coagulation cascade, and increase capillary permeability [39,40]. Anticoagulant proteins such as Thrombomodulin (TM), endothelial cell protein C receptor, and tissue fibrinogen activator (tPA) are produced by endothelial cells but are significantly reduced in PE pregnancies [41]. Activated Protein C on the endothelial surface may lose its anticoagulant function, exposing tissue factor, and primary coagulation activator [41]. The protein SERPINA5 has been found to inhibit these anticoagulant factors [42]. Additionally, SERPINA5 may play a role in cancer biology [42]. SERPINA5 suppresses breast cancer cell proliferation, metastasis, angiogenesis, and cell invasion by decreasing urokinase (uPA) and enhancing breast cancer cell adhesion [42,43]. SERPINA5 has been identified as an inhibitor of anticoagulant protease-activating protein C, which inhibits the proteases of coagulation and fibrinolysis, including thrombin, and the thrombin-coagulant complex, factor Xa [42,44,45]. SERPINA5 also regulates the healing process by inhibiting the activity of the hepatocyte growth factor activator [46]. Our previous in vitro study found that SERPINA5 inhibits human EVT invasion by reducing uPA protein activity and decreasing MSP expression [15,16]. This evidence supports the hypothesis that SERPINA5 overexpression in rats may induce PE-like features.

Our in vitro studies suggest that inhibiting SERPINA5 expression enhances EVT invasion, potentially ameliorating PE features. We aimed to determine whether reducing SERPINA5 expression in vivo could disrupt PE progression. Given rats’ relatively brief gestation period, the timeframe for trophoblast invasion is limited. Consequently, we pre-injected ADV to suppress SERPINA5 expression. ADV expression typically begins 2–3 days post-injection, ensuring reduced SERPINA5 expression before L-NAME injection. Our findings suggest that the pre-inhibition of SERPINA5 expression partially alleviates PE-like features in rats, as evidenced by reduced hypertension, proteinuria, and improved placental development. However, symptoms of fetal developmental restriction persisted, possibly due to the initiation of SERPINA5 inhibition via an adenovirus-based method only on the fifth day of pregnancy. Therefore, the onset of SERPINA5 inhibition may have been delayed relative to the impact of L-NAME. A Cochrane meta-analysis of 77 studies involving 40,249 women indicated that low-dose aspirin reduced the incidence of PE by 18 percent (95% CI, 12–23%) [47]. In line with these findings, our research suggests that SERPINA5 inhibition attenuates coagulation cascade activation and may improve PE prognosis.

This study has several limitations. Caution is required when examining the pathogenic or therapeutic roles of SERPINA5 using ADV, as it may complicate symptom differentiation. Future research should consider using targeted gene knock-in or knock-out methods. The placental weights reported in this study represent the total weight of the obtained tissues, which were not perfused. While each graph presents data from six tissues per experimental group, these values may exceed those reported in other studies. Furthermore, the levels of SERPINA5 in pregnancy are still confusing, emphasizing the need for longitudinal studies to assess SERPINA5 levels throughout the gestational period.

## 5. Conclusions

In conclusion, our research indicates that SERPINA5 may be a predisposing factor for PE. Additionally, our findings suggest that SERPINA5 in the placenta could serve as a potential therapeutic target to enhance the prognosis of PE.

## Data Availability

All data generated or analyzed during this study are available from the corresponding author upon reasonable request.
